# Supplementary material for: Measuring the impact of the Capital Card®, a novel form of contingency management, on substance misuse treatment outcomes: A retrospective evaluation
Source: PLoS One. 2020 Mar 10;15(3):e0229905. doi: 10.1371/journal.pone.0229905 (PMC7064232; doi:10.1371/journal.pone.0229905)
Supplement: Attachment — Submitted filename: CC Plos ONE Response to Reviewers.docx [file pone.0229905.s001.docx]

**Response to Reviewers**

We have provided detailed responses to each of the reviewers’ comments and suggestions, below. We are grateful for these comments as they have improved our manuscript in a number of key areas, and we hope that our responses and revisions are acceptable.

**Reviewer #1**

This is an unmasked review of a manuscript principally describing retrospective analysis of a Capital Card, likened by the authors to a commercial loyalty card, and its association with substance use treatment outcomes over a two-year period among 1545 persons from a United Kingdom borough. A limited set of demographic data were examined and controlled for in later analyses examining differences in treatment outcomes between those entering treatment programs in the year prior vs. following introduction of the Capital Card. Reported findings note that, after controlling for age and substance of abuse, those entering treatment programs following introduction of the Capital Card were more likely than their counterparts to complete treatment. While a primary study caveat (lack of randomization to comparison groups) is acknowledged by the submitting authors, it carries significant limitations and prevents meaningful contribution to extant addiction treatment literature. In particular, this quasi-experimental design precludes any control for likely influences of history as well as a host of other 3rd-variables (socioeconomic status, income, addiction severity, and treatment facility to name a few) oddly omitted from consideration despite author indication that all persons included in the analyses had completed a comprehensive intake assessment. Additional study caveats include: 1) inadequate description of the service data from which the categorical determination of treatment outcome occurred; 2) errant description of the intention of the Capital Card as a form of voucher-based contingency management, when in fact its description as a point system instead reflects something much more like a type of token economy; 3) absence of behavioral reinforcement for a large majority of Capital Card recipients, as evidenced by report that 85+% of Capital Card recipients never exchanged their ‘points’ for any tangible reinforcer, that calls into question whether this Capital Card functioned as a form of contingency management; and 4) the included suggestion of an ethically questionable provision that during the study period Capital Card recipients were concurrently recruited to promote use of the Capital Card ‘scheme’ to others.

**RESPONSE**: We are grateful to this reviewer for their constructive feedback on our manuscript. On specific points raised by the reviewer we have the following responses/amendments to report:

*“In particular, this quasi-experimental design precludes any control for likely influences of history as well as a host of other 3rd-variables (socioeconomic status, income, addiction severity, and treatment facility to name a few) oddly omitted…”.* The reviewer is quite right to highlight the absence of these factors which would likely impact on outcomes. However, none of these data were captured in the standardised assessment, and so were unavailable for inclusion in the analysis. With particular regard to ‘treatment facility’, we would note that these data were based upon a single London Borough, with services all delivered by the same provider (WDP), so the use of facility as a variable would not be appropriate. With regards to the other variables, we have now explicitly highlighted their absence in the Discussion.

*“…errant description of the intention of the Capital Card as a form of voucher-based contingency management, when in fact its description as a point system instead reflects something much more like a type of token economy”* and *“…absence of behavioral reinforcement for a large majority of Capital Card recipients…”.* We thank the reviewer for these astute observations, which we admit we had not considered in our analysis. While we maintain that the Capital Card has itself, as a complex intervention, been designed as a form of contingency management, it is indeed the case that during the window in which we have conducted our analysis, it has been used as something more akin to a token economy. We have commented on this important point in our discussion, indicating that future work over a longer period of follow up would be necessary to establish both whether service users ever in fact spend the majority of their points, or whether the token economy-type interaction remains the norm.

*“the included suggestion of an ethically questionable provision that during the study period Capital Card recipients were concurrently recruited to promote use of the Capital Card ‘scheme’ to others”*. We do not agree that the involvement of service users in the ongoing development promotion of the scheme is ethically questionable, but appreciate that our very brief description of this aspect of the scheme may have left this open to such an interpretation. The role and nature of ambassadors has been further clarified in the manuscript, along with an example of the positive feedback received from ambassadors, who are provided with support and training, which is intended to help develop further transferable employment skills. Ultimately, the involvement of services users as champions and ambassadors has been central to the development of the Capital Card scheme, to ensure that the way the scheme operates reflects the needs of those who use it.

**Reviewer #2**

This report describes a retrospective assessment of the effectiveness of an incentive program used in outpatients with substance use disorders. Outcomes obtained with 429 patients treated during the year after the incentive program was implemented were compared to those from 1,116 treated during the year before implementation (overall N= 1,545) in a single borough in London. After controlling for sample differences in socio-demographic characteristics and primary substance use disorder, odds of completing treatment were 1.5 times greater in the incentives compared to no-incentives conditions. Acknowledging the limitation of the research design for supporting causal inferences, the authors reasonably conclude that these results are sufficiently positive to warrant conducting a randomized controlled trial to prospectively assess the effectiveness of this incentive intervention.

As the authors appropriately note in the Introduction, dissemination of Contingency Management (CM) interventions into community substance abuse treatment services has been disappointing considering the enormous amount of empirical evidence from controlled studies supporting its efficacy. For that reason, the present report detailing what appears to be a successful implementation of CM into community treatment programs has the potential to be of interest to researchers, policy makers, and clinicians involved with prevention and treatment of substance use disorders. The report is generally well written, and the study appears to have been reasonably well conducted, and the results analyzed and interpreted appropriately. Enthusiasm is dampened to some extent by a lack of information in several areas detailed below. None of these matters are necessarily fatal flaws, but they should be addressed.

(1) The authors assume a familiarity with the service provider, WDP. I took the initiative to investigate online but readers should not need to do so. Please revise the opening sentence of the Abstract keeping in mind that many readers of this international journal will have no familiarity with WDP, nor “sector, first awards”, and may not understand what you mean by the parenthetical mention of the NICE clinical guideline on CM. Please revise or deleate that sentence. Please also add a couple of sentences to the Methods that provides readers with additional information on WDP and the services it provides. I can see why the authors may want to mention the sector first award, but that should only be mentioned once, either in the Intro or Discussion and with sufficient background for readers to understand its significance.

**RESPONSE**: we have amended both the abstract and introduction section as suggested.

(2) Please add additional detail to the Methods on the following three aspects of the CM intervention. Say more using examples of they type of activities that earned points. Note the monetary value of points, monetary value of total possible earnings, whether number of points that could be earned varied by activity, and if possible the average monetary value of incentives earned in the present study. Lastly, please provide more information regarding the arrangement that WDP has with local retailers to provide incentives. Is the incentive program entirely funded through charitable donations from these retailers? Are NICE funds involved and if not, why not? Lastly, please move to the Methods the information that is now in the Discussion regarding patients being able to spend points for 12 months following treatment completion and to continue earning them for an additional 6 months. Those are important features of the program.

**RESPONSE**: We thank the reviewer for these points, which we agree are important to provide clarity on the nature of the scheme and how it operates in practice. We have given a more extensive list of the types of activities for which points can be earned. Attaching a monetary value to points is not possible, as the scheme is not designed in this way (i.e. with a fixed monetary value per point). However, we have provided some examples of the number of points which would be required for some specific activities, to give readers a clearer idea of what is available, and what number of points is required for different activities. Finally, we have provided more detailed information on the funding model which underpins the operation of the Capital Card Scheme.

(3) Please provide additional breakdown of outcomes by primary type of SUD. You may consider adding an interaction term to the model on type of SUD. Even a descriptive breakdown on treatment completion rates before and following implementation of the incentive program by primary SUD would be helpful. Readers are surely going to be curious about potential differences.

**RESPONSE**: We have included a table showing successful completion rates, by primary substance, across both cohorts, and also overall. We thank the reviewer for highlighting this, as it is important information and provides the reader with a sense of the treatment outcome profiles by primary substance.

(4) Please add parenthetical percentages to the tables so readers don’t have to do the calculations on their own as they read through the report, which is what I found myself doing.

**RESPONSE**: we have now added percentages as suggested.
